# Enhanced biomedical applicability of ZrO_2_–SiO_2_ ceramic composites in 3D printed bone scaffolds

**DOI:** 10.1038/s41598-022-10731-w

**Published:** 2022-04-27

**Authors:** Chih-Hao Chang, Chih-Yang Lin, Chih-Hung Chang, Fwu-Hsing Liu, Yu-Tzu Huang, Yunn-Shiuan Liao

**Affiliations:** 1 Department of Orthopedics, National Taiwan University Hospital and National Taiwan University College of Medicine, No.7, Chung Shan S. Rd., Zhongzheng Dist., Taipei, 100225 Taiwan, ROC; 2 National Taiwan University Hospital Jin-Shan Branch, No.7, Yulu Rd., Wuhu Village, Jinshan Dist., New Taipei, 20844 Taiwan, ROC; 3 Department of Mechanical Engineering, National Taiwan University, No. 1, Sec. 4, Roosevelt Rd., Taipei, 10617 Taiwan, ROC; 4 Department of Orthopedic Surgery, Far Eastern Memorial Hospital, No.21, Sec. 2, Nanya S. Rd., Banciao Dist., New Taipei, 22000 Taiwan, ROC; 5 Graduate School of Biotechnology and Bioengineering, Yuan Ze University, No.135, Yuan-Tung Road, Zhongli Dist., Taoyuan, 32003 Taiwan, ROC; 6 Department of Mechanical Engineering, LungHwa University of Science and Technology, No.300, Sec.1, Wanshou Rd., Guishan Dist., Taoyuan, 333326 Taiwan, ROC; 7 College of Medicine, Fu Jen Catholic University, No.300, Sec.1, Wanshou Rd., Guishan Dist., Taoyuan, 333326 Taiwan, ROC

**Keywords:** Engineering, Materials science

## Abstract

Zirconia (ZrO_2_) has been widely used in clinical applications, such as bone and dental implantation, because of its favorable mechanical properties and resistance to fracture. However, the poor cell affinity of ZrO_2_ for bone regeneration and tissue binding, as well as its shrinkage due to crystal phase transformation during heat treatment, limits its clinical use and processing plasticity. This study aims to investigate an appropriate ZrO_2_–SiO_2_ composite recipe for ceramic 3D printing processes that can strike a balance between the mechanical properties and cell affinity needed in clinical applications. Specimens with different ZrO_2_–SiO_2_ composite recipes were fabricated by a selective laser gelling method and sintered at temperatures ranging from 900 to 1500 °C. The S5Z5 composite, which consists of 50 wt% ZrO_2_, 35 wt% SiO_2_ and 15 wt% SiO_2_ sol, showed an appropriate compressive strength and bending strength of 82.56 MPa and 55.98 MPa, respectively, at a sintering temperature of 1300 °C. The shrinkage rate of the S5Z5 composite was approximately 5% when the sintering temperature was increased from 900 to 1500 °C. All composites exhibited no cytotoxicity after 144 h of MG63 cell incubation, and the S5Z5 composite exhibited the most obvious cell affinity among the composite recipes. From these results, compared with other composites, the S5Z5 composite was shown to possess mechanical properties and a cell affinity more comparable to those of natural human bone.

## Introduction

The clinical demand for the orthopedic treatment of large bone defects has been increasing over the years because of increased rates of osteoporosis and critical accidents. Currently, bone regeneration therapy employs autografts or allografts to enhance bone healing in large bone defects^1,2^. Natural bone is often used for implantation since it provides an ideal environment for cell growth, thereby shortening the bone regeneration period. However, patients may experience adverse immunological reactions, which increased the risk of contracting transmissible diseases via implants. In addition, the quantity of available natural bone implants is insufficient to meet the demands of clinical surgery^3^. Accordingly, tissue engineering (TE), including the engineering of cells, growth factors, and scaffolds, has been explored to overcome these aforementioned difficulties. Scaffolds are of special importance since they provide an environment for cellular adhesion, proliferation, and differentiation^4,5^. The materials of scaffolds must possess appropriate mechanical properties to ensure structural stability during bone reconstruction^6,7^. Hence, the selection of scaffold materials and the approaches for fabricating scaffolds remain crucial research topics^8^.

Metals, polymers, ceramics, and composite materials are common scaffold materials used in clinical applications^9^. Metals can offer sufficient support to maintain the structure of a scaffold, but their surfaces need to be modified to enhance their bioactivity^10,11^. Polymers have good flexibility similar to that of natural bone, but their rapid degradation and material weakness limit their applicability^12–14^. Ceramics possess good mechanical properties and cell affinity, which make them suitable for cell growth^15–17^. In particular, ZrO_2_ has long been used in clinical applications because of its mechanical properties and resistance to fracture^18,19^. However, ZrO_2_ is not bioactive and cannot chemically or biologically bond to bones^20^. The low cell affinity together with the high thermoshrinkage of ZrO_2_ may limit its application in scaffold manufacturing and medical utilization^21^. On the other hand, SiO_2_ has excellent cell and tissue affinities via the interaction of its silanol group with the calcium and phosphate ions in biological fluids^22,23^. Studies have demonstrated that ZrO_2_ can react with SiO_2_ in the liquid phase to form ZrO_2_–SiO_2_ compounds^24^. These ZrO_2_–SiO_2_ compounds have shown good biological properties suitable for medical applications since they have the ability to release silicate ions that facilitate the growth and differentiation of osteoblasts^25^. However, the influence of the ZrO_2_–SiO_2_ composition on the mechanical properties of these compounds was not investigated in these previously reported studies.

Ideal bone scaffolds should have highly interconnected porous structures that can induce the formation of bone from the surrounding tissue or act as a template for growing cells for bone tissue regeneration^26–28^. However, it has been very difficult to manufacture this desired structure until the advent of 3D printers. 3D printing technology can facilitate the fabrication of complex structures based on a layer-by-layer principle^29–31^. Hung et al. used a fused deposition manufacturing (FDM) process and employed polyurethane/hyaluronan/TGFβ3 to form soft scaffolds that promoted the self-aggregation of mesenchymal stem cells (MSCs) and induced the chondrogenic differentiation of MSCs to produce a matrix for cartilage repair^32^. Wang et al. used sodium alginate/polyvinyl formal composite as a raw material to fabricate porous scaffolds for bone remolding^33^. Weinand et al. used hydrogels as a binder to fabricate porous β-TCP scaffolds with a 3D printing method, which led to better regeneration of bone tissue with MSCs^34^. Lee et al. used binder jetting to build polycaprolactone/chitosan bone scaffolds with an interconnected structure that facilitated the spreading and proliferation of MSCs after an apatite-coating treatment^35^. A selective laser gelling (SLG) process for the manufacturing of 3D parts called ceramic laser gelling (CLG) was developed^36^ and applied to fabricate a CaCO_3_–SiO_2_ interporous bioceramic scaffold^37^. Near-zero volume shrinkage of the specimens after sintering was realized since inorganic materials (SiO_2_ sol in this case) were used as the binder. In another study, when an inorganic binder was used in the SLG process, the volume shrinkage was found to be lower than that observed when an organic binder was used in other 3D printing processes^38^.

The compressive strength of CaCO_3_–SiO_2_ composites in a previous study was 47 MPa, but this is much lower than that of human bone, which is 100–230 MPa^39^. Hence, the objective of this study was to determine an appropriate bioceramic material and sintering temperature that could improve the mechanical properties of and impart satisfactory biological properties to specimens prepared by the SLG process. ZrO_2_–SiO_2_ compounds have been suggested to have these desired properties and therefore were selected for this study.

To achieve the required properties, various ZrO_2_–SiO_2_ composite recipes were designed, and specimens manufactured by the SLG process were sintered at various temperatures^40^. Their mechanical properties, microstructures, and cell affinities were investigated. X-ray diffraction (XRD) analysis was conducted for a detailed interpretation of the experimental results. Finally, the most appropriate recipe was applied to fabricate a biomimetic bone scaffold, and its feasibility in clinical applications was assessed.

## Results

### Mechanical properties

**Figure 1:**
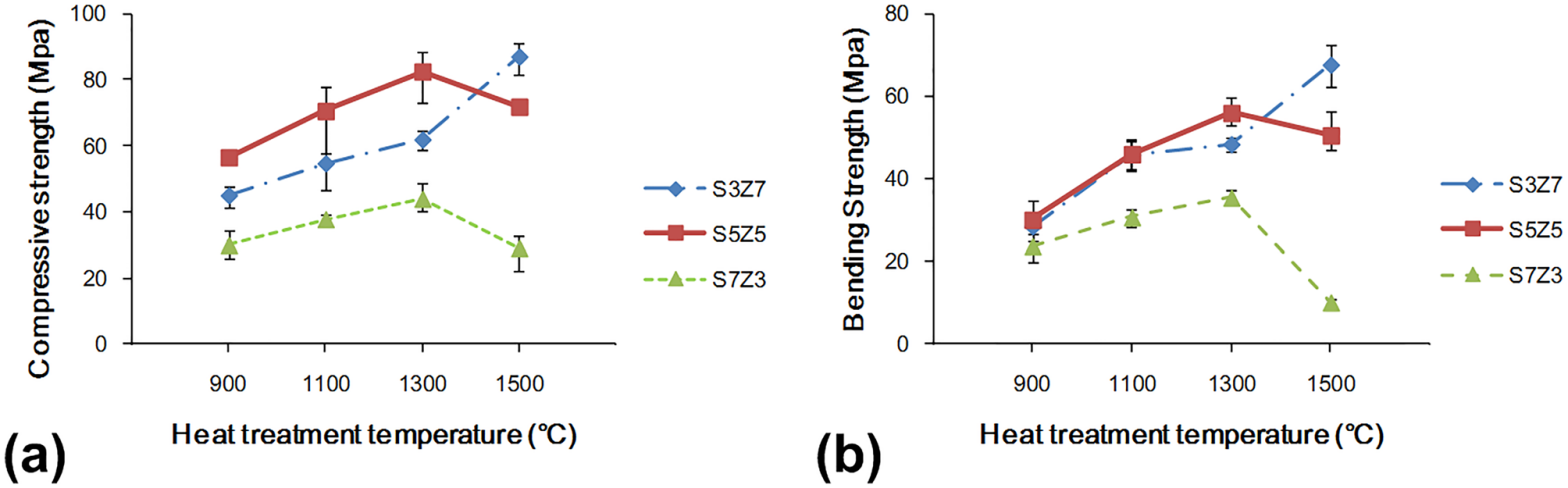
Mechanical properties of the S3Z7, S5Z5 and S7Z3 specimens after heat treatment at 900 °C, 1100 °C, 1300 °C and 1500 °C: (**a**) compressive strength, and (**b**) bending strength.

**Figure 2:**
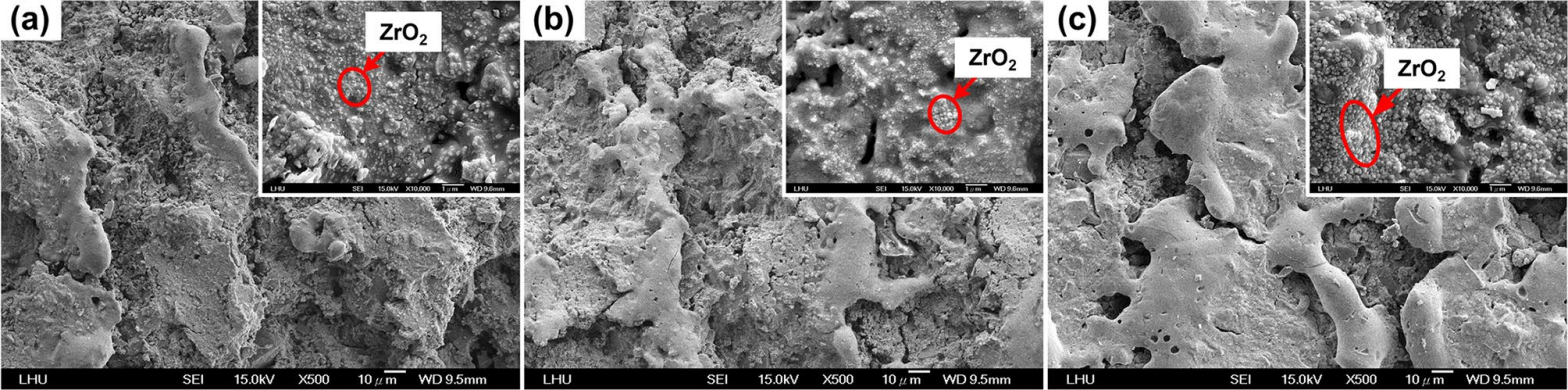
Surface topography images of S5Z5 specimens with low magnification (× 500) and high magnification (× 10,000, shown in the upper right corner) after heat treatment at (**a**) 900 °C, (**b**) 1100 °C and (**c**) 1300 °C.

**Figure 3:**
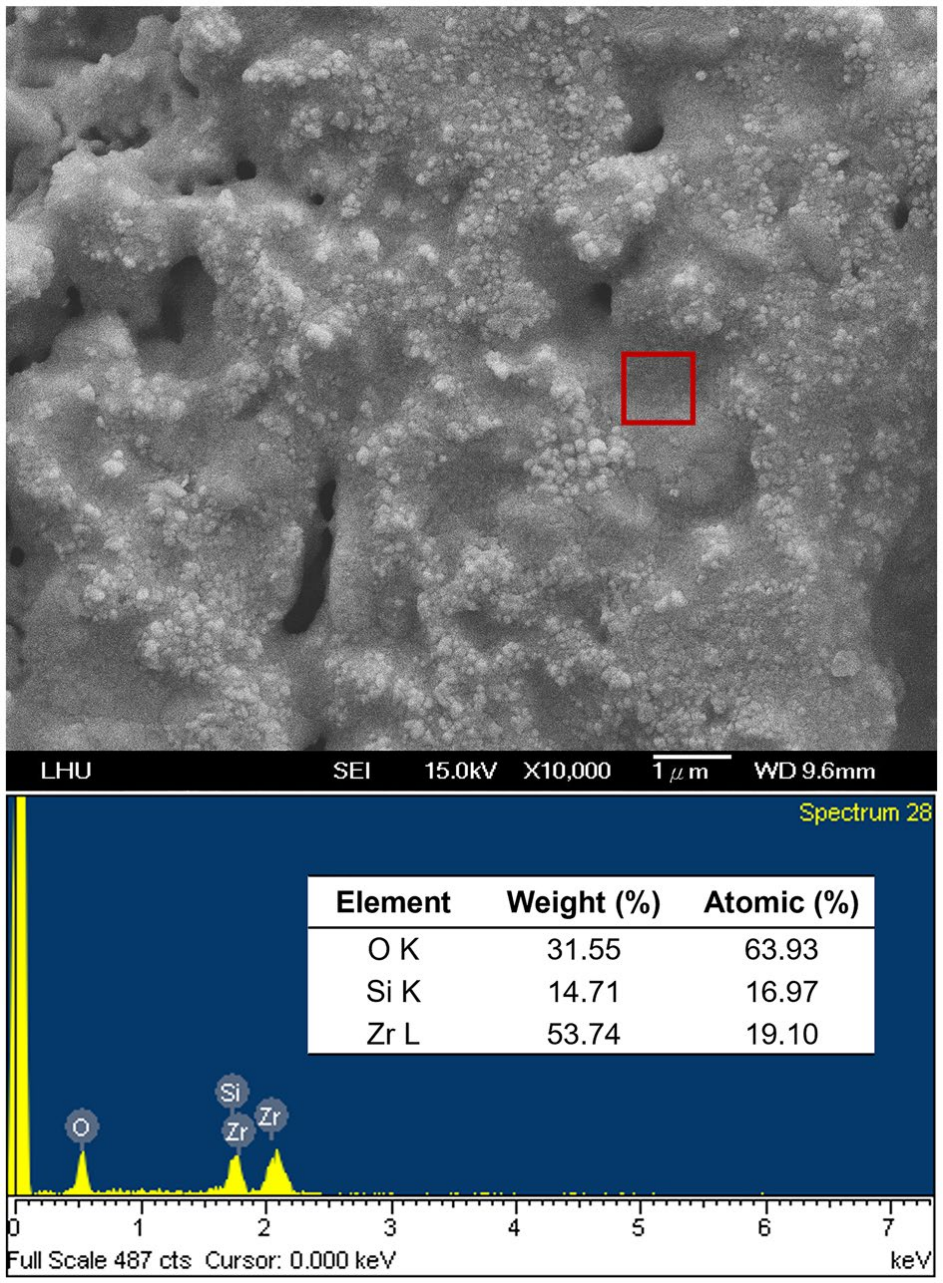
EDX elemental analysis of the S5Z5 specimen after heat treatment at 1100 °C.

To understand the relationship between the mechanical properties induced by various ZrO_2_–SiO_2_ recipes and the heat treatment temperatures, the compressive and bending strengths of the samples prepared by three different recipes at different temperatures of heat treatment were determined, and the results are shown in Fig. 1. Both the compressive strength and bending strength increased with the heat treatment temperature in the range of 900–1300 °C. The recrystallization temperature of gelled SiO_2_, which is between 800 and 1200 °C, depended on the manufacturing process. The gradual recrystallization of gelled SiO_2_ increased with increasing temperature, which could strengthen the binding force between particles and lead to better mechanical properties. When the sintering temperature was raised from 1300 to 1500 °C, the mechanical properties of S3Z7 were enhanced, but those of S5Z5 and S7Z3 deteriorated. Figure 2 shows the low magnification (500×) SEM images of S5Z5 at heat treatment temperatures of 900 °C, 1100 °C, and 1300 °C. The corresponding high magnification (10,000×) SEM images shown in the upper right corners were also observed. The recrystallization of gelled SiO_2_ with increasing temperature was verified by observing the obvious melting of gelled SiO_2_ on the specimen. From the high-magnification SEM microphotographs, the grain size of ZrO_2_ increased with increasing temperature. Oh proposed that the recrystallization of gelled SiO_2_ would further improve the mechanical properties of ZrO_2_–SiO_2_ compounds^41^. In addition, SEM and EDX elemental analyses of S5Z5 at a heat treatment temperature of 1300 °C were conducted, as shown in Fig. 3. The selected frame indicates the region of ZrO_2_ particles in recrystallized SiO_2_, and the weight ratios of O, Si and Zr were 31.6%, 14.7% and 53.7%, respectively.

**Figure 4:**
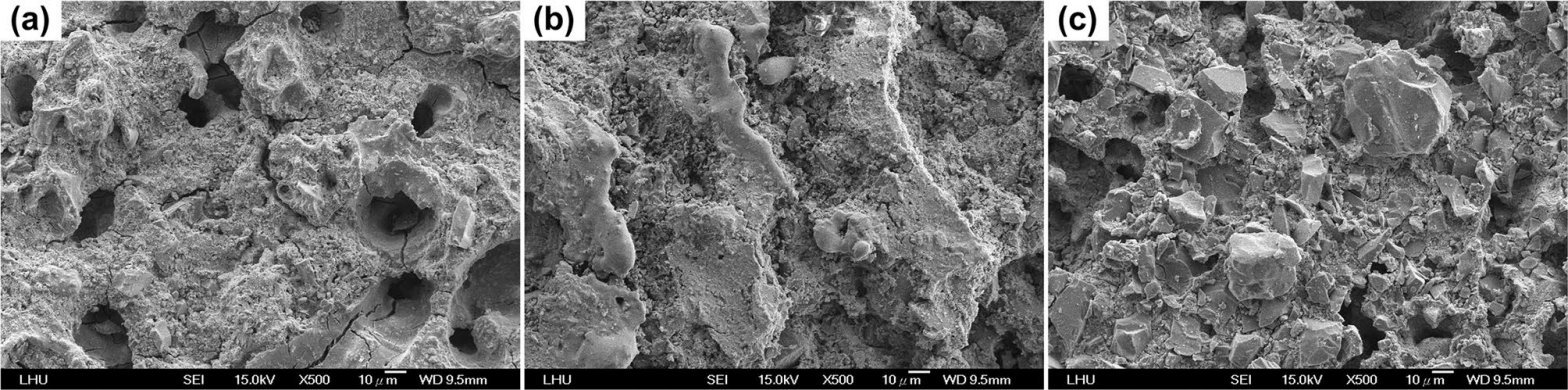
Surface topography images of specimens after heat treatment at 900 °C: (**a**) S3Z7, (**b**) S5Z5 and (**c**) S7Z3.

**Figure 5:**
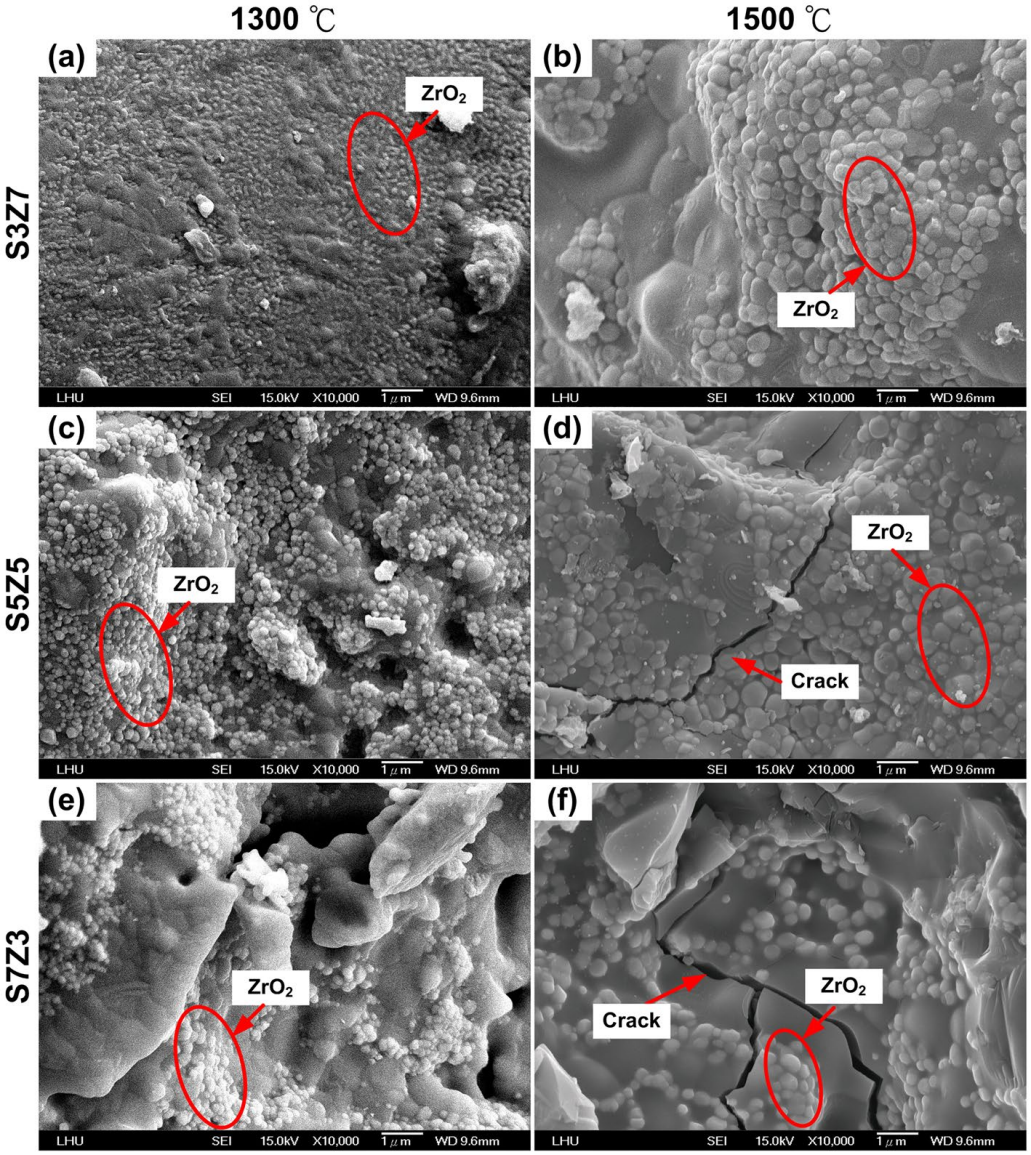
Surface topography images of S3Z7 (**a**,**b**), S5Z5 (**c**,**d**) and S7Z3 (**e**,**f**) specimens after heat treatment at 1300 °C (**a**,**c**,**e**) and 1500 °C (**b**,**d**,**f**).

The low-magnification (500×) SEM microphotographs of S3Z7 and S7Z3 sintered at 900 °C were compared to that of S5Z5, as shown in Fig. 4. In Fig. 4a, many cracks on the surface of S3Z7 can be observed, as well as a dense microstructure with many micropores on the surface. No obvious cracks were observed on the surfaces of S5Z5 and S7Z3, as shown in Fig. 4b,c. High-magnification (10,000×) SEM microphotographs of specimens prepared by three recipes at heat treatment temperatures of 1300 °C and 1500 °C are shown in Fig. 5. There were no microcracks on the surfaces of the specimens prepared by three recipes for a heat treatment temperature of 1300 °C. Once the sintering temperature was increased to 1500 °C, noticeable microcracks appeared on the surfaces of S5Z5 and S7Z3, while no such cracks were found on the surface of S3Z7.

**Figure 6:**
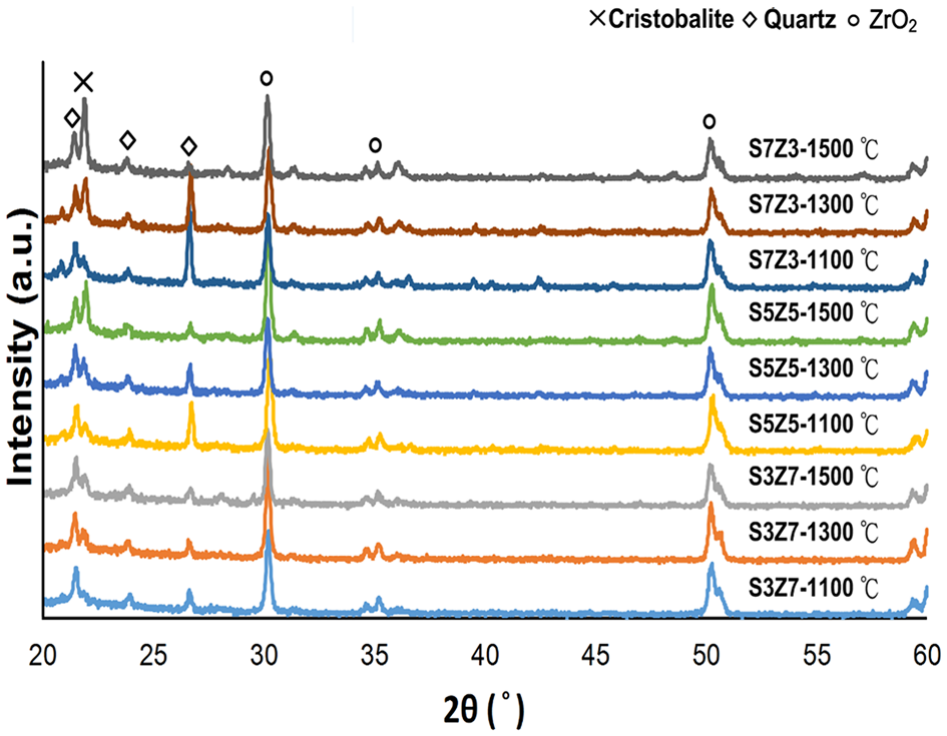
XRD results of the S3Z7, S5Z5 and S7Z3 specimens after heat treatment at 1100 °C, 1300 °C and 1500 °C.

The XRD results of the specimens prepared by three recipes at sintering temperatures of 1100 °C, 1300 °C, and 1500 °C are shown in Fig. 6. The cristobalite peaks (2θ = 22°) of S5Z5 and S7Z3 increased significantly, while the peaks of the quartz phase (2θ = 26.6°) disappeared almost completely when the temperature was increased to 1500 °C^42,43^. As a result, the density was reduced from 2.5 to 2.3 g/cm^3^, and expansion of SiO_2_ occurred accordingly^44^. The transformation of quartz to cristobalite occurred over a temperature interval from approximately 1300 °C up to the melting point of SiO_2_ at 1710 °C, and the transformation from quartz to an intermediate amorphous state reached a maximum at 1677 °C. From XRD analysis, the phase transformation from quartz into cristobalite was observed in the specimens prepared by three recipes, especially in S5Z5 and S7Z3, over a temperature range of 1100 to 1500 °C during heat treatment.

Based on an analysis of the resulting mechanical properties, S7Z3 was eliminated as a potential candidate material since it showed insufficient compressive and bending strengths. The S3Z7 and S5Z5 specimens were heat treated at 1300 °C and 1500 °C, respectively, and were kept for further consideration based on the shrinkage of the fabricated specimens.

### Porosity and volume shrinkage/expansion

**Figure 7:**
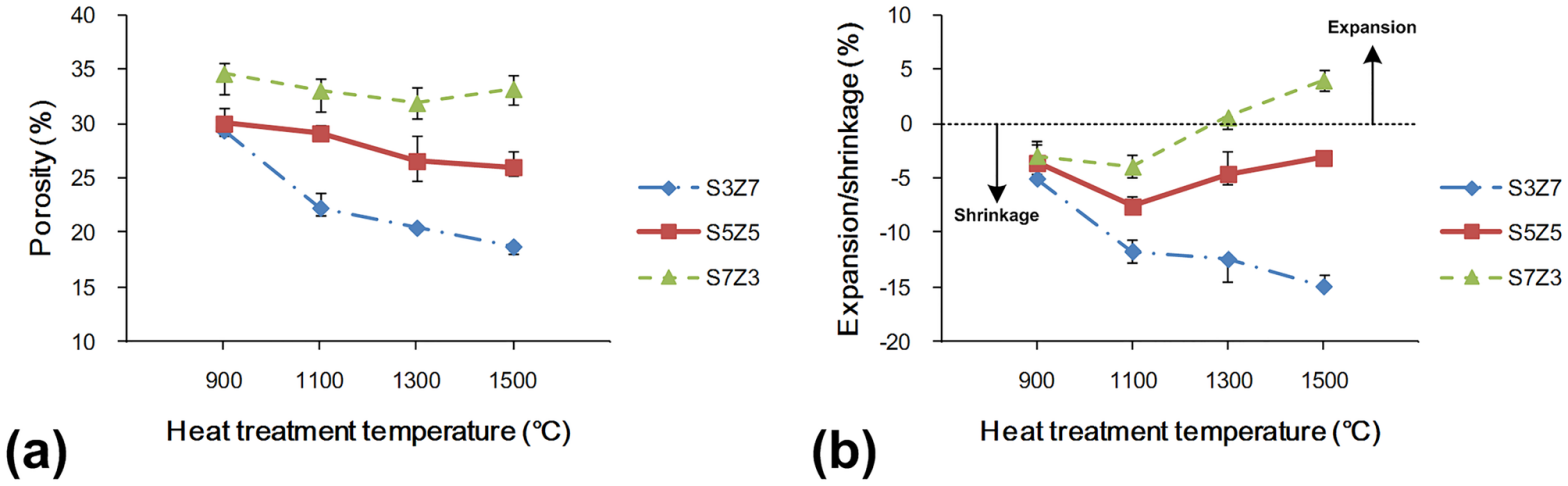
(**a**) Porosity and (**b**) volume shrinkage of the S3Z7, S5Z5, and S7Z3 specimens after heat treatment at 900 °C, 1100 °C, 1300 °C and 1500 °C.

Figure 7 shows the changes in porosity and volume for S3Z7, S5Z5, and S7Z3 after sintering at different temperatures. The porosities of the three recipe samples decreased with increasing temperature, but only S7Z3 exhibited a slight increase in porosity at 1500 °C. It was also noted that the variation in porosity for S5Z5 was insignificant over the temperature range of 1300–1500 °C. Figure 7b shows the changes in volume with different temperatures. The volume shrinkage of S3Z7 increased with increasing temperature. However, for S5Z5 and S7Z3, the shrinkage only increased in the temperature range of 900–1100 °C and decreased above 1100 °C. Moreover, instead of shrinkage, an expansion was observed for S7Z3 at 1500 °C. From SEM analysis, it was observed that the agglomeration of ZrO_2_ powder took place at all temperatures, as depicted in Fig. 2. This agglomeration was greater for the composite with a higher ZrO_2_ content that was treated at a higher temperature. Since the size of the SiO_2_ powder particles was far larger than that of the ZrO_2_ powder particles, the spaces between the SiO_2_ powder particles would be filled by the ZrO_2_ powder particles and their agglomerates. This action, together with the recrystallization of the SiO_2_ gel, caused the densification of the specimen structure and a reduction in the specimen porosity, as shown in Fig. 7a. The small variations in the porosities of S5Z5 and S7Z3 in the temperature range of 1300–1500 °C will be explained in a later section. As shown in Fig. 5, the cristobalite peak could be seen for all composites at all temperatures used in this study. Hence, an increase in the volume was expected for all composites under any temperature, and the extent of volume expansion was expected to depend on the SiO_2_ content. Therefore, S7Z3 showed the largest volume increase among the three composites. The increase in volume could compensate for the reduction in porosity discussed previously, and it led to small variations in the porosities of S5Z5 and S7Z3 over the temperature range of 1300–1500 °C. The greater size of ZrO_2_ at higher temperatures, as discussed before, also contributed to the increase in the volume. On the other hand, the volume was reduced by the agglomeration of the ZrO_2_ powder particles. This action dominated the volume change of S3Z7 for all investigated temperatures. S5Z5 and S7Z3 showed an affected volume change over the temperature range of 900–1100 °C. However, starting at 1300 °C, the volume increased because the reasons discussed above became more significant, and hence, the behavior was exhibited as shown in Fig. 7b.

### In vitro test

**Figure 8:**
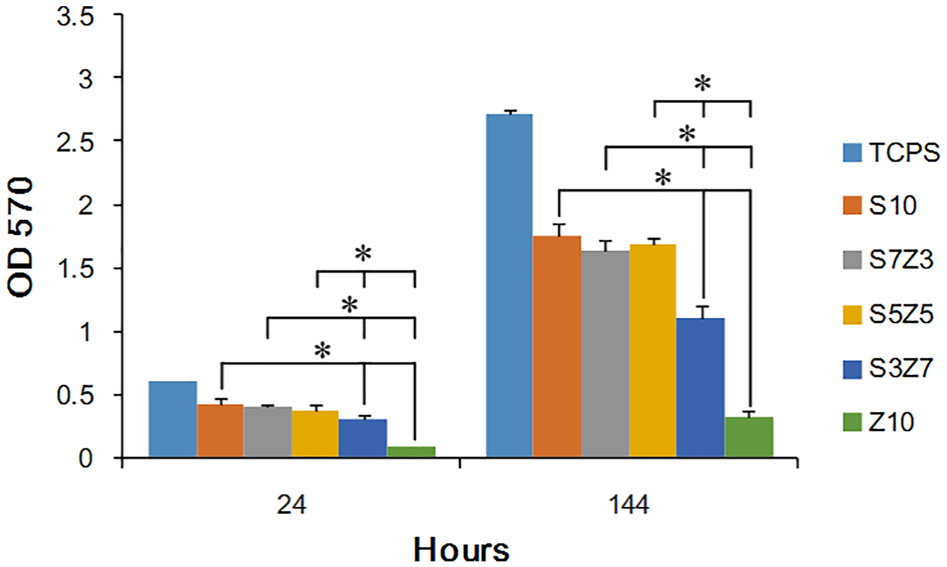
Cell adhesion and growth of MG63 cells on the S10, S3Z7, S5Z5, S7Z3 and Z10 specimens. Statistical analysis was performed by Student’s t test on the values obtained from five independent specimens. The level of significance was established at *P < 0.05.

After optimizing the thermoshrinkage and mechanical strength of the ZrO_2_–SiO_2_ composites, the samples after heat treatment at 1300 °C were used for further cell affinity and proliferation tests. Figure 8 shows the proliferation of MG 63 cells in terms of the OD values of the MTT assay on the S10, S7Z3, S5Z5, S3Z7, and Z10 samples after 24 h and 144 h of culturing. As shown in the figure, S10 (pure SiO_2_) and Z10 (pure ZrO_2_) were used as the positive and negative controls, respectively, to determine if the cell affinity would be affected by the specific compositions of the ZrO_2_–SiO_2_ recipes. The OD values were detected for all samples after 24 h of cell culturing, indicating the initial adherence of the cells to the samples. Significantly, cell growth was observed after a long culturing time of 144 h for all the samples, and hence, the proliferation of the cells on the ZrO_2_–SiO_2_ samples could be considered evident and satisfactory. It was also noted from the figure that S5Z5 showed the best cell proliferation.

**Figure 9:**
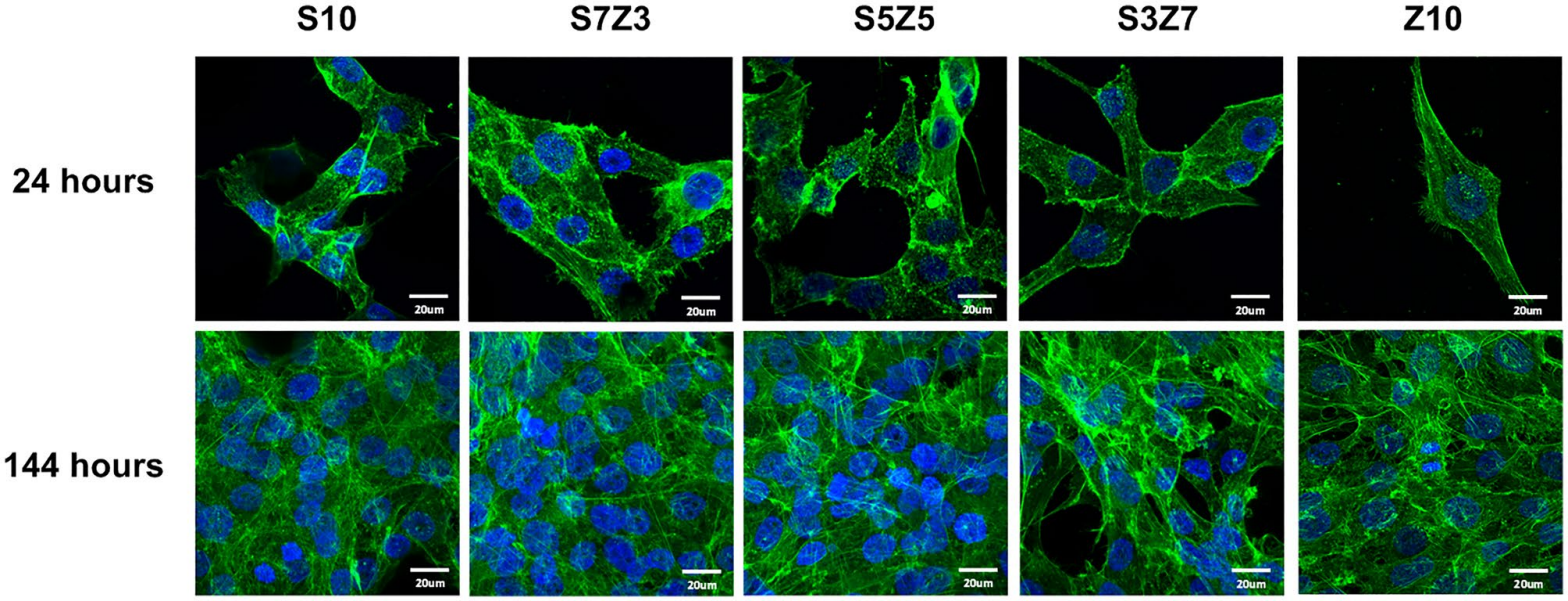
Cell fluorescence staining of the S10, S3Z7, S5Z5, S7Z3 and Z10 specimens. Green signals indicate actin, and blue signals indicate the nuclei of cells.

A fluorescent stain was used to assess the state of cell adhesion by checking if the cells maintained their original shape, and the results are shown in Fig. 9. From the MTT assay, it was found that MG63 cells were attached to all the specimens. The morphology of the cells changed to a spindle-shaped morphology after 24 h of culturing, implying that ordinary growth of the cells had occurred^45^. The number of MG63 cells increased, and the actin of MG63 connected with the surrounding cells when the culturing time was extended to 144 h.

### Fabrication of a biomimetic bone scaffold

**Figure 10:**
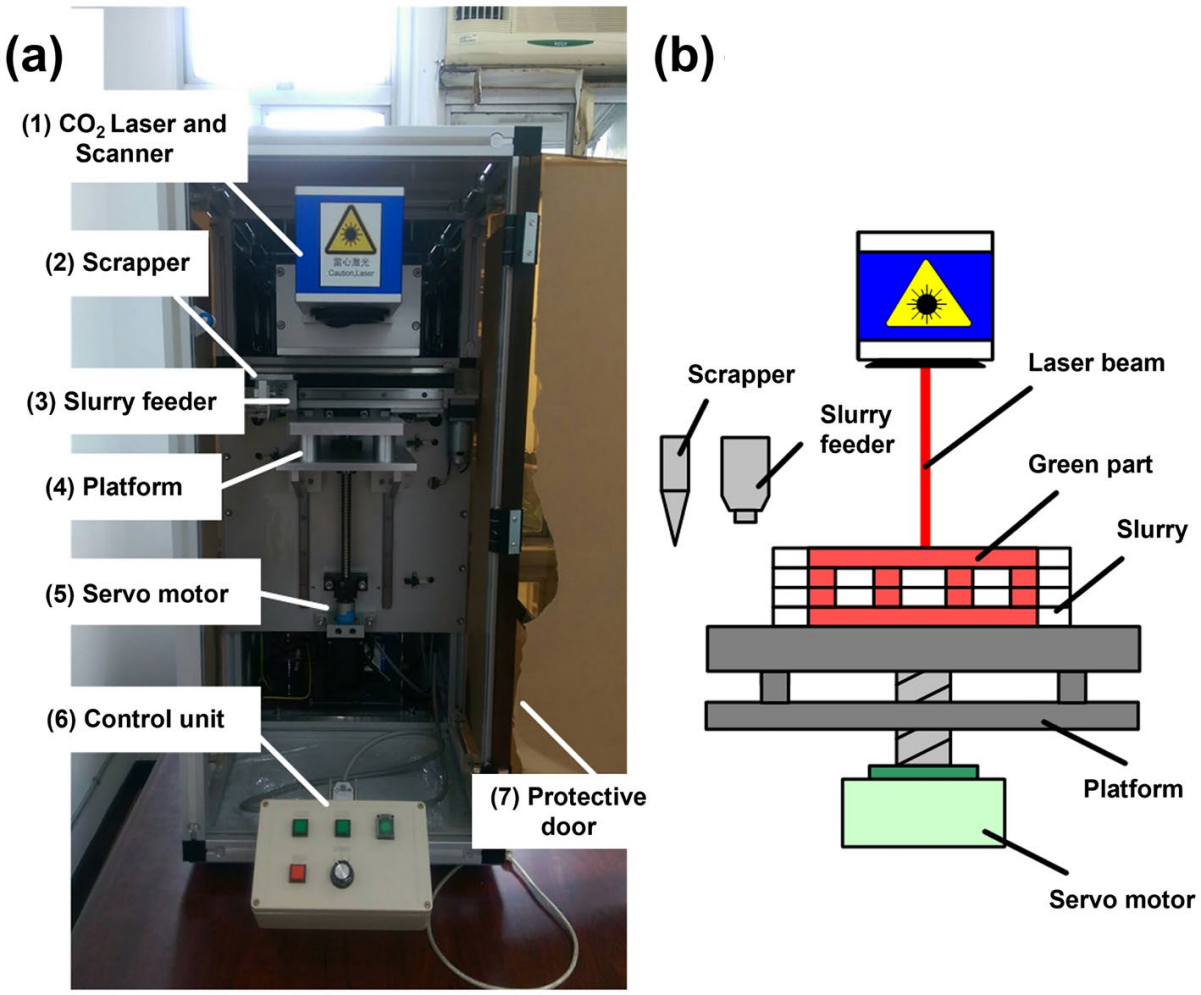
Schematic of the (**a**) 3D printing machine and (**b**) laser-aided gelling method.

**Figure 11:**
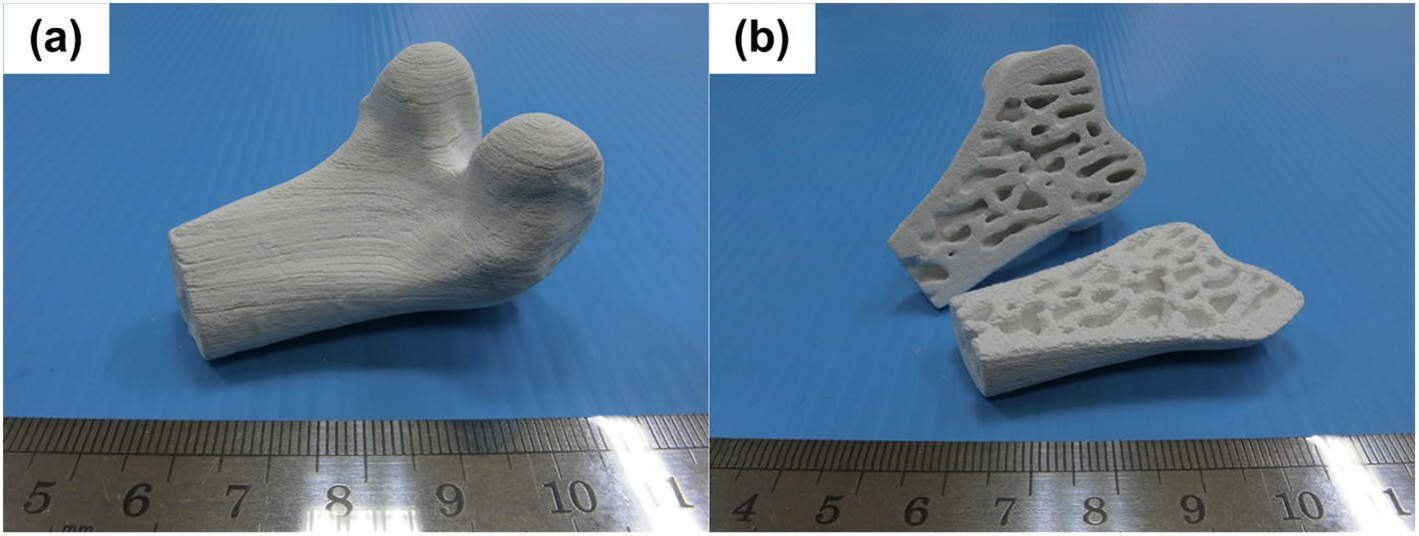
Bioceramic bone scaffold with a complex structure: (**a**) isometric view, and (**b**) cross section.

The S5Z5 composite was employed to manufacture a biomimetic bone scaffold because its properties of thermoshrinkage, compressive strength, and cell affinity were better than those of S3Z7 and S7Z3 when the green parts of the fabricated ceramic composite specimens were heat-treated at a temperature of 1300 °C. The digital files of the outer shape and inner structure for the scaffold were transformed and merged by software for the fabrication process, as shown in Fig. 10. Figure 11 shows the biomimetic bone scaffold (the size is approximately W 35 mm × H 30 mm × L 50 mm) produced by the SLG technique. From the figure, the complex outer and inner structures of the bone model were successfully reconstructed. This indicates that a scaffold with a complex structure made of the desired material S5Z5 can indeed be fabricated by the SLG method.

## Discussion

This study aimed to investigate whether an appropriate ZrO_2_–SiO_2_ bone graft manufactured by the SLG process could result in improved mechanical properties and impart satisfactory biological properties for clinical use. As a result of analyzing the compressive and bending strengths at different sintering temperatures, it was observed that the compressive and bending strengths of S7Z3 were lower than those of S5Z5 and S3Z7 in the temperature range of 900–1300 °C during heat treatment. Compared to the other composites, S7Z3 exhibited the worst mechanical properties and more microcracks on the surface. Additionally, the microstructure of agglomerated ZrO_2_ in S7Z3 was not as dense as that in S5Z5 and S3Z7. In Fig. 5, recrystallized SiO_2_ is more obvious than agglomerated ZrO_2_ in S7S3 compared to S5Z5 and S3Z7. This phenomenon was due to the difference in the particle sizes and weight ratios of ZrO_2_ and SiO_2_. The average particle sizes of ZrO_2_ and SiO_2_ were 0.1 μm and 25 μm, respectively, and the specific surface areas of S3Z7 and S5Z5 were higher than that of S7Z3. Therefore, the microstructure of agglomerated ZrO_2_ in S7Z3 was not as dense as that in S5Z5 and S3Z7 after sintering. The reduction in the mechanical strength due to the formation of micropores within the structure affected the mechanical support provided by ZrO_2_. The existence of Zr, Si, and O ions may indicate the formation of a ZrO_2_–SiO_2_ compound from the solid reaction of ZrO_2_ and SiO_2_ (powder and/or sol)^24^. This solid reaction could enhance the bonding strength between the two different materials^46^. Similar ZrO_2_–SiO_2_ compounds were found for all three composites at various heat treatment temperatures. The formation of ZrO_2_–SiO_2_ compounds was found to be a temperature-dependent reaction, which could facilitate an improvement in the mechanical properties of the composites with increasing temperature. The maximum compressive and bending strengths of pure SiO_2_ were 33.6 and 26.2 MPa after heat treatment at 1300 °C. In comparison, the maximum compressive and bending strengths of the three composites, especially S5Z5, were higher than those of pure SiO_2_. From the SEM analysis, it was observed that there was no obvious damage on the surface of S5Z5. The microstructure of S3Z7 was dense with many micropores on the surface. The reason for this could be the higher specific surface area of the ZrO_2_ powder compared to that of the SiO_2_ powder. The effect of the reduction in the mechanical strength of the structure due to the formation of micropores was larger than the effect of the improvement in the mechanical properties of the structure owing to ZrO_2_. As a result, the compressive and bending strengths of S3Z7 were lower than those of S5Z5. For S7Z3, its microstructure was not as dense as that of S3Z7. Furthermore, there were microcracks on its surface. The formation of microcracks played a major role in the reduction of the compressive and bending strengths. As shown in Fig. 5, obvious microcracks appeared on the surfaces of S5Z5 and S7Z3 when the sintering temperature was increased to 1500 °C, while no such cracks were found on the surface of S3Z7. The microcracks on the surfaces of S5Z5 and S7Z3 could be generated by the expansion of SiO_2_ because of its phase transformation at a high temperature. A similar transformation of amorphous SiO_2_ to cristobalite, which influenced mechanical properties, has also been reported by He et al.^47^.

According to the results, large volume shrinkage and small porosity were observed for S3Z7, which was not considered an appropriate composite for the 3D printing of bone scaffolds for medical applications. The S7Z3 composite was excluded due to its unsatisfactory mechanical properties, as described in “3D printer and manufacturing procedures”. For S5Z5, although there was a small difference in its shrinkage and porosities at sintering temperatures of 1300 °C and 1500 °C, its mechanical properties were much better at 1300 °C. The volume shrinkage of S5Z5 was only 5%, with a satisfactory porosity of 26.57%. Hence, S5Z5 with a heat treatment temperature of 1300 °C was considered the better composite for the SLG process.

Based on the results of the MTT assay and fluorescence staining, it was observed that none of the specimens exhibited cytotoxicity. A lower cytotoxicity of a material could provide better environments for cellular adhesion, spread, and proliferation. From Fig. 8, it can be seen that the OD values of the MTT assay for S5Z5 and Z10 were 1.6 and 0.3, respectively, after 144 h of culturing. S5Z5 showed the best cell affinity among the composites, especially compared to ZrO_2_ (i.e., Z10 in the test), which has long been used in clinical applications (such as dental implants and ball heads of femoral implants)^48^. Only for ZrO_2_ was the reduction of cell proliferation reduced greatly. Various ZrO_2_–SiO_2_ compounds for the manufacturing of bone scaffolds by the SLG method were investigated in this study. S5Z5 shows more suitable mechanical properties and cell affinity for manufacturing artificial bone scaffolds for medical implantation, and its introduction into the bone-repair market can be expected in the near future.

## Methods

### 3D printer and manufacturing procedures

The 3D printer used in this study was self-designed and assembled, as shown in Fig. 10a. It consists of the following devices: (1) a CO_2_ laser and scanner, (2) scrapper, (3) slurry feeder, (4) platform, (5) servo motor, and (6) control unit. In the 3D printing process, the platform moves to its starting position first by the action of the servo motor. This is followed by slurry injection through the slurry feeder. Next, the scraper paves the slurry to ensure a uniform layer of a specific thickness. Then, the platform moves downward along the layer thickness, and at the same time, the slurry feeder and scraper return to their original positions. The whole procedure is repeated until the green part of the ceramic composite specimen is finished. A schematic of the building process for the green part of a ceramic composite specimen on the platform is shown in Fig. 10b. Once the green part is fabricated, it is sintered in a furnace (P310, Nabertherm, Germany) to improve its mechanical properties.

### Materials and selective laser gelling

ZrO_2_ powder, SiO_2_ powder, and SiO_2_ sol were used as raw materials for the SLG process. The ZrO_2_ and SiO_2_ powders acted as fillers to reinforce the structures of the fabricated specimens, while the SiO_2_ sol was used as a binder in the construction of the bioceramic green parts of the fabricated specimens. The powder sizes of ZrO_2_ and SiO_2_ were 0.1 μm and 25 μm, respectively. The SiO_2_ sol consisted of 60 wt% water and 40 wt% SiO_2_ nanoparticles that were 40 nm in size. The ZrO_2_ and SiO_2_ were mixed in various proportions so that the appropriate material composition resulting in the best mechanical properties and cell affinity could be obtained. Three recipes were tested, as shown in Table 1. The composites prepared by these three recipes are denoted as S3Z7, S5Z5, and S7Z3 according to the weight percentages of SiO_2_ and ZrO_2_ in the compounds. The ceramic slurries containing the ZrO_2_ powder, SiO_2_ powder, and SiO_2_ sol were further blended by ball milling with a rotational speed of 100 rpm for 1 h to ensure that they were mixed homogeneously.

**Table 1:** Contents of ZrO_2_-based SiO_2_ ceramic composites.

	ZrO_2_ powder (wt%)	SiO_2_ powder (wt%)	SiO_2_ sol (wt%)
S3Z7	70	15	15
S5Z5	50	35	15
S7Z3	30	55	15

The principle of part fabrication in this study is based on the irreversible sol–gel reaction of the SiO_2_ sol. Coagulation of SiO_2_ leading to gelling takes place because of the change in the pH or moisture evaporation as a result of the dissolution of the –OH terminus of the silicic acid (Si(OH)_4_) of SiO_2_ sol, which generates a condensation reaction^49^. Hence, the region not irradiated by a CO_2_ laser can be readily removed by deionized water, and complex structures, such as undercuts, overhangs, or inner channel structures, can be fabricated by the SLG process^29^.

The specimens used for evaluating the compressive strength, bending strength, volume shrinkage, and porosity of the ZrO_2_–SiO_2_ compounds were fabricated by using the following process parameters: a laser power of 7 W, laser scanning speed of 100 mm/s, laser scanning hatch of 0.1 mm, and layer thickness of 0.1 mm. The compressive and bending strengths of the specimens were tested in accordance with the JIS R1608 and JIS R1601 standards, respectively. The specimens used for the compressive strength test were shaped as disks that were 6 mm in diameter and 12.5 mm in height, and the specimens used for the bending strength test were shaped as cuboids that were 30 mm in length, 4 mm in width, and 3 mm in height. The dimensions of the specimens used for evaluating the porosity and volume shrinkage were 10 mm × 10 mm × 4 mm. To determine the appropriate sintering temperature, the testing specimens were sintered at 900 °C, 1100 °C, 1300 °C and 1500 °C for 2 h with a heating rate of 5 °C/min.

### Analysis of mechanical and physical properties

A universal test machine (HT-9102, Hungta, Taiwan) was used to test the average compressive and bending strengths of the fabricated specimens according to JIS R1608 and JIS R1601. The maximum values of the compressive and bending strengths were determined by the first inflection points of the compression and bending curves, respectively. Archimedes’ principle was used to evaluate the porosity of the specimens, and the numerical value of the porosity was calculated as follows:

1 $$ {\text{Porosity}}\,(\% ) = \frac{{{{w_{2}  - w_{3}  - w_{s} } \mathord{\left/ {\vphantom {{w_{2}  - w_{3}  - w_{s} } {\rho _{e} }}} \right. \kern-\nulldelimiterspace} {\rho _{e} }}}}{{{{w_{1}  - w_{3} } \mathord{\left/ {\vphantom {{w_{1}  - w_{3} } {\rho _{e} }}} \right. \kern-\nulldelimiterspace} {\rho _{e} }} + {{w_{s} } \mathord{\left/ {\vphantom {{w_{s} } {\rho _{s} }}} \right. \kern-\nulldelimiterspace} {\rho _{s} }}}} \times 100, $$

where ${\rho }_{e}$ and ${\rho }_{s}$ are the densities of ethanol and the specimen used in the test, respectively. The terms ${w}_{1}$, ${w}_{2},$ ${w}_{3}$, and ${w}_{s}$ are the weight of the beaker filled with ethanol, the weight of the beaker containing ethanol and the specimen, the combined weight of the beaker and the remaining ethanol after the ethanol-filled specimen is taken out of the beaker, and the weight of the specimen, respectively^50^. To evaluate the volume shrinkage, a digital microscope (VHX-2000, Keyence, USA) was used to measure the dimensions of the fabricated specimen, and then the volume changes as a percentage before and after heat treatment were calculated. Three specimens were tested for each condition, and crystalline phase analysis of the specimens was conducted by X-ray diffraction (XRD) analysis (D/Max 2200, Rigaku, Japan) with CuKα radiation. Each test was performed for 2θ values between 20° and 60° at a step size of 5°/min. The topographies, elemental compositions, and crystalline structures of the specimens were observed using scanning electron microscopy (SEM) and energy-dispersive X-ray spectrometry (EDX) (JSM-6500f, JEOL, Japan).

### Test of the cell affinity and proliferation

The cell affinity of the ZrO_2_–SiO_2_ composites was tested by inoculating MG63 cells (10^4^ cells/well) directly onto the surface of the cuboid specimens (4.5 mm × 4.5 mm × 1 mm) in a 96-well tissue-culture plate. The cell-culture temperature was kept at 37 °C with a CO_2_ concentration of 5%. MG63 proliferation was determined using the MTT (3-[4,5-dimethylthiazol-2-yl]-2,5 diphenyl tetrazolium bromide) assay with an ELISA reader (VersaMax, Molecular Devices, USA) to measure the optical density (OD 570 nm). Cell adhesion and spreading were observed using stereomicroscopy (SEM-1500, Nihon, Japan) and imaged (Pro-150ES, Pixera, Japan) after 24 h and 144 h of culturing, respectively. The ceramic specimens were washed with PBS and fixed with 2.5% paraformaldehyde at 25 °C. After the cells were fixed, the nuclei and actin of the MG63 cells were stained at 37 °C with DAPI (FluoroPure™ Grade) and phalloidin (Fluor 488) for 5 and 10 min, respectively. Five specimens were used for the biological tests.

### Fabrication of a biomimetic bone scaffold

To manufacture a biomimetic bone scaffold, a digital file of the scaffold’s outer shape was obtained by scanning a human femur bone model with a 3D scanner (Comet 5, Carl Zeiss Optotechnik GmbH, Germany). A digital file of the scaffold’s inner structure was obtained by scanning a cancellous bone model using μCT (Skyscan 1176, Bruker, Belgium). These two digital files were transformed into STL files by the Mimics software and merged by the Magics software. The manufacturing parameters were input into the developed machine for the fabrication process, as shown in Fig. 10. The S5Z5 composite was employed for manufacturing the biomimetic bone scaffold, and the green part of the composite after the SLG process was heat-treated at 1300 °C.
